# Global Surgery at the National Landscape: Perspectives after the XXXIV Brazilian Congress of Surgery

**DOI:** 10.1590/0100-6991e-20223189

**Published:** 2022-03-11

**Authors:** LETÍCIA NUNES CAMPOS, LUCAS SOUSA SALGADO, SOFIA WAGEMAKER VIANA, ARISTÓCLES HÍTALLO BEZERRA, ASHER MISHALY, LÍVIA SOUSA RIBEIRO, ANGELA THERESA ZUFFO YABRUDE, CAROLINE MARQUES DE AQUINO, RODRIGO VAZ FERREIRA, JÚLIA LOYOLA FERREIRA, FABIO BOTELHO

**Affiliations:** 1 - International Student Surgical Network of Brazil - Brasil; 2 - Universidade de Pernambuco, Faculdade de Ciências Médicas - Recife - PE - Brasil; 3 - União Educacional do Vale do Aço, Faculdade de Medicina - Ipatinga - MG - Brasil; 4 - Kursk State Medical University, Faculdade de Medicina - Kursk - Kurskaya Oblast - Rússia; 5 - Centro Universitário Unifacisa, Faculdade de Medicina - Campina Grande - PB - Brasil; 6 - Universidade Nove de Julho, Faculdade de Medicina - São Paulo - SP - Brasil; 7 - Universidade Federal do Recôncavo da Bahia, Centro de Ciências da Saúde - Santo Antônio de Jesus - BA - Brasil; 8 - Universidade Regional de Blumenau, Faculdade de Medicina - Blumenau - SC - Brasil; 9 - Universidade do Estado do Amazonas, Disciplina de Cirurgia - Manaus - AM - Brasil; 10 - McGill University - Montreal - Quebec - Canadá; 11 - Montreal Children’s Hospital, McGill University Health Center, Harvey E. Beardmore Division of Pediatric Surgery - Montreal - Quebec - Canadá

**Keywords:** Global Health Strategies, Health Equity, Quality Indicators, Health Care, Health Workforce, Estratégias de Saúde Globais, Equidade em Saúde, Indicadores de Qualidade em Assistência à Saúde, Mão de Obra em Saúde

## Abstract

The XXXIV Brazilian Congress of Surgery included Global Surgery for the first time in its scientific program. Global Surgery is any action in research, clinical practice, and policy-making that aims to improve access and quality of care in surgical specialties. In 2015, The Lancet Commission on Global Surgery highlighted that five billion people lack safe, timely, and affordable surgical care. Even more critical, nine of ten people cannot access essential surgical care in low and middle-income countries, where a third of the worldwide population resides, and only 6% of global surgical procedures are performed. Although Brazilian researchers and institutions have been contributing to lay the movement’s foundations since 2014, Global Surgery remains a barely debated subject in the country. It is urgent to expand the field and break paradigms regarding the surgeons’ role in public health in Brazil. Accomplishing these standards requires a joint effort to strategically allocate resources and identify collaboration opportunities, including those from medical societies and regulatory bodies. As members of the International Student Surgical Network of Brazil - a nonprofit organization by and for students, residents, and young physicians focused on Global Surgery - we review why investing in surgery is cost-effective to strengthen health systems, reduce morbimortality, and lead to economic development. Additionally, we highlight and propose key recommendations to foster the field at the national level.

## RESUMO

O 34° Congresso Brasileiro de Cirurgia incluiu Cirurgia Global pela primeira vez em seu programa científico. Cirurgia Global é qualquer ação em pesquisa, prática clínica e políticas em saúde que visa melhorar o acesso e a qualidade do atendimento em especialidades cirúrgicas. Em 2015, a Comissão da The Lancet em Cirurgia Global destacou que cinco bilhões de pessoas carecem de assistência cirúrgica segura, oportuna e acessível. Ainda mais crítico, nove em cada dez pessoas não têm acesso a cuidados cirúrgicos essenciais em países de baixa e média renda, onde um terço da população mundial reside e apenas 6% dos procedimentos cirúrgicos globais são realizados. Embora pesquisadores e instituições brasileiras tenham contribuído para lançar as bases internacionais e nacionais do movimento desde 2014, a Cirurgia Global ainda é um assunto pouco debatido no país. Assim, faz-se urgente expandir essa área de conhecimento e quebrar paradigmas quanto ao papel do cirurgião na saúde pública no Brasil. Isso requer um esforço conjunto para alocar recursos de forma estratégica bem como para identificar oportunidades de colaboração, incluindo as sociedades médicas e os órgãos reguladores. Como membros da *International Student Surgical Network of Brazil* - organização sem fins lucrativos feita por e para estudantes, residentes e jovens médicos com foco na Cirurgia Global - revisamos por que investir em Cirurgia é uma medida custo-efetiva para fortalecer os sistemas de saúde, reduzir a morbimortalidade e promover o desenvolvimento econômico. Além disso, destacamos e propomos recomendações-chave para fomentar a Cirurgia Global a nível nacional.

## INTRODUCTION

The XXXIV Brazilian Congress of Surgery started on the 2^nd^ of September 2021 and highlighted different tools to facilitate the daily clinical-surgical practice of general surgeons. Amidst round tables and panels, the almost centenary Brazilian College of Surgeons included Global Surgery as a topic for the first time with the session “Global Surgery: Promoting Equity in Access to Surgical Care”. Indeed, Global Surgery is an emerging and seldom debated subject in our country, even though Brazilian researchers and institutions have been contributing to lay the movement’s international and national foundations since 2014. Seven years later, there is still an urgency to expand Global Surgery and break paradigms regarding the surgeons’ role in public health in Brazil.

But, what is Global Surgery? Global Surgery is any action in research, clinical practice, and policy-making that aims to improve access and quality of care in surgical specialties such as trauma, anesthesia, and obstetrics^1^ ^,^ ^2^. In 2015, The Lancet Commission on Global Surgery highlighted that five billion people lack safe, timely, and affordable surgical care. Even more critical, nine of ten people cannot access essential surgical care in low- and middle-income countries - (LMICs), where a third of the world’s population resides and only 6% of global surgical procedures are performed^1^ ^,^ ^2^. Consequently, 1.27 million additional surgeons, anesthesiologists, and obstetricians are required by 2030 to cover this gap and foster healthcare worldwide^1^ ^,^ ^3^. Moreover, low operative volumes are associated with high case-fatality rates from common, treatable surgical conditions, with 143 million additional surgical procedures needed in LMICs each year to save lives and prevent disability^1^. 

Besides the reduction in morbidity and mortality, investing in surgical care is cost-effective. Without increased funding in surgical scale-up, LMICs will hamper economic productivity and lose an estimate of US$12,3 trillion by 2030^1^. Hence, empowering surgical healthcare leads to economic development and social change. Accomplishing such standards requires a joint effort to strategically allocate resources and identify collaboration opportunities, including those from medical societies and regulatory bodies^4^. Furthermore, investments in surgical training, curricula development, and workforce distribution are crucial steps to promote emergency and elective surgical care^4^. 

Effective integration of Global Surgery-related competencies into medical education and surgical practice remains a challenge, especially in medical schools and residency programs. Upon this point, there is no universal set of academic Global Surgery skills to inform educational programs. Moreover, high-income countries’ institutions publish most of the literature, a natural bias to address the complexity of different settings and populations^5^. 

Brazil still has numerous challenges in healthcare despite the existence of a comprehensive public health system. While regions such as the North have approximately 20 surgeons, anesthesiologists, and obstetricians per 100.000 inhabitants, the minimal expected specialist surgical workforce density, the South easily surpasses 40 professionals per 100.000 inhabitants^6^. It is worth mentioning that such estimates may mask the inequitable distribution of surgical providers in the states from the same geographical region. This heterogeneous distribution can be explained by the lack of salary stability, infrastructure, and continuity to improve surgical training^7^. Another hazard is related to the higher density of professionals in the private sector, while 71,5% of Brazilians depend exclusively on the public health system^7^. Also, the burden of diseases is changing in Brazil, without being followed by the Ministry of Health priority agenda. Even though infectious diseases are no longer the leading causes of mortality, they remain a priority for funding and policies. In contrast, surgical conditions, such as trauma, remain the leading cause of morbidity and do not appear on the public health agenda^6^. 

Immediate interventions are vital to change the current scenario. The simplest one is to expand the knowledge of Global Surgery’s importance and applicability in our country. The International Student Surgical Network (InciSioN) is a worldwide nonprofit organization by and for students, residents, and young physicians focused on Global Surgery with more than 5,000 members spread across 70 countries, including Brazil. As national representatives of InciSioN Brazil, we welcome the initiative to include Global Surgery in the conference schedule. Nevertheless, we hope that such a milestone does not remain exclusive to academic conferences but expands to a long-term commitment of medical societies and educational institutions. In addition, it is essential to mention that developing Global Surgery at the national level will place Brazil in a prominent position to lead the movement as a country with one of the most robust publicly funded health care systems. Thus, we propose the following recommendations to foster Global Surgery in Brazil:

1. Support and propose bilateral collaboration with the current Global Surgery groups, including trainee-led organizations, to raise advocacy within surgeons, anesthesiologists, obstetricians, and the community;
2. Encourage medical societies of various surgical specialties to produce locally adapted learning resources and promote capacity-building about Global Surgery in their conferences, seminars, and other educational events;
3. Insert Global Surgery on medical schools’ curricula and as a component of surgical, anesthesia, as well as obstetrics and gynecology residency programs;
4. Establish a research priority agenda concerning the surgical diseases that most aggravate the health of Brazilians for the next five years;
5. Guarantee a diverse and inclusive panel of Global Surgery stakeholders to analyze, implement, and monitor research and health policies based on Brazilian population needs;
6. Academic institutions should provide supervision, intellectual and financial support to students willing to work with the established priorities;
7. Based on the findings, propose interventions and public health policies in the Regional Health Commissions and National Health Council;
8. Renew the priority plan and policies every five years.

Investing in surgery is the best cost-effective action to decrease inequities and promote development. Surgery is not as complex or expensive as commonly believed^1^. It consists of a sustainable approach to improve access to healthcare as well as strengthen our surgical workforce, funding, infrastructure, resources, and services at the national level. More than aiding on the “how to treat” theme of the latest Brazilian Congress of Surgery under a patient-surgeon perspective, Global Surgery can serve as a pillar to enhance the role of surgeons to tackle health inequities within our Unified Health System.
